# Vascular function assessed with cardiovascular magnetic resonance predicts survival in patients with advanced chronic kidney disease

**DOI:** 10.1186/1532-429X-10-39

**Published:** 2008-08-18

**Authors:** Patrick B Mark, Arthur Doyle, Kevin G Blyth, Rajan K Patel, Robin AP Weir, Tracey Steedman, John E Foster, Henry J Dargie, Alan G Jardine

**Affiliations:** 1 BHF Glasgow cardiovascular research centre, faculty of medicine, University of Glasgow, Glasgow, Scotland, G12 8TA, UK; 2 Renal unit, Western infirmary, Glasgow, Scotland, G11 6NT, UK; 3 Department of cardiology, Western infirmary, Glasgow, Scotland, G11 6NT, UK

## Abstract

**Background:**

Increased arterial stiffness is associated with mortality in patients with chronic kidney disease. Cardiovascular magnetic resonance (CMR) permits assessment of the central arteries to measure aortic function.

**Methods:**

We studied the relationship between central haemodynamics and outcome using CMR in 144 chronic kidney disease patients with estimated glomerular filtration rate <15 ml/min (110 on dialysis). Aortic distensibilty and volumetric arterial strain were calculated from cross sectional aortic volume and pulse pressure measured during the scan.

**Results:**

Median follow up after the scan was 24 months. There were no significant differences in aortic distensibilty or aortic volumetric arterial strain between pre-dialysis and dialysis patients. Aortic distensibilty and volumetric arterial strain negatively correlated with age. Aortic distensibilty and volumetric arterial strain were lower in diabetics, patients with ischaemic heart disease and peripheral vascular disease. During follow up there were 20 deaths. Patients who died had lower aortic distensibilty than survivors. In a survival analysis, diabetes, systolic blood pressure and aortic distensibilty were independent predictors of mortality. There were 12 non-fatal cardiovascular events during follow up. Analysing the combined end point of death or a vascular event, diabetes, aortic distensibilty and volumetric arterial strain were predictors of events.

**Conclusion:**

Deranged vascular function measured with CMR correlates with cardiovascular risk factors and predicts outcome. CMR measures of vascular function are potential targets for interventions to reduce cardiovascular risk.

## Introduction

Premature cardiovascular disease is the leading cause of death in patients with end stage renal disease (ESRD) treated with dialysis. The relationship between cardiovascular (CV) risk factors and CV events is less clear in ESRD than in the general population, with paradoxical relationships between both cholesterol and blood pressure and CV risk[1,2]. The presence of increased arterial stiffness is a predictor of CV morbidity and mortality in ESRD[3,4]. Conventionally, this has been assessed by measurement of pulse wave velocity (PWV). More recently, cardiovascular magnetic resonance (CMR) has been used to identify tissue abnormalities in the heart of ESRD patients[5]. This imaging modality also permits contemporaneous visualisation of large arteries and direct measurement of aortic function, providing an integrated assessment of both ventricular and vascular function in one examination. Aortic distensibilty (AD), the relative change in aortic size throughout the cardiac cycle relative to blood pressure, is readily assessed with CMR. Given the strong relationship between vascular stiffness and outcome in ESRD, CMR measures of vascular function may identify patients at increased CV risk. We have demonstrated in a previous pilot study using CMR that aortic stiffness is increased in patients with ESRD[6]. More recently aortic stiffness has been demonstrated with CMR to be increased in patients with even mild chronic kidney disease (CKD), equivalent to findings in patients with heart failure with preserved systolic function[7].

To date however, the relationship between aortic distensibilty (AD), as measured with CMR and long term outcome has not been assessed. Thus, we assessed the relationship between CMR measures of vascular function and conventional CV risk factors in ESRD patients and studied the long term prognostic implications of CMR measures of vascular function.

## Methods

### Subjects

Studies were performed on 144 patients with chronic kidney disease (CKD) stage 5[8]. All patients were assessed as part of a CV screening program for renal transplantation and therefore were either on dialysis therapy or expected to start dialysis within six months. All patients gave written, informed consent and the study was approved by the local ethics committee.

### CMR technique

**Figure 1:**
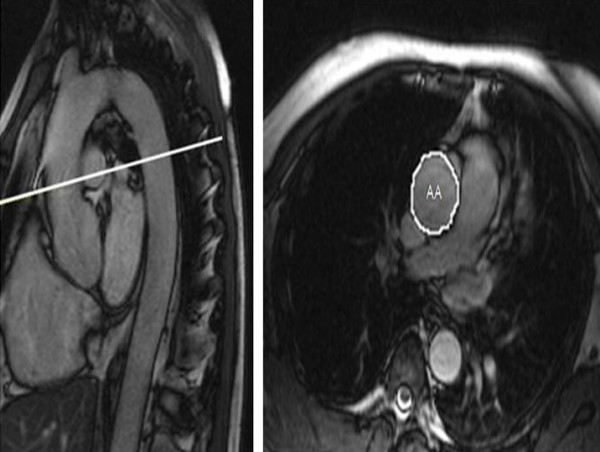
Representative images demonstrating sagittal view (left) used to plan transverse plane aortic images (right) with schematic tracing of ascending aorta (AA).

CMR was performed using a 1.5 Tesla MR scanner (Sonata, Siemens, Erlangen, Germany). In haemodialysis patients imaging was performed on the post-dialysis day whereas peritoneal dialysis patients were studied at their "dry weight". Aortic volume was acquired from cine CMR images in the transverse plane of the ascending aorta, obtained at the level of the main pulmonary artery, using a steady-state free precession (true FISP) sequence, TR = 3.2 ms, TE = 1.6 ms, FA = 60°, FoV 276 × 340 mm, pixel dimensions 2.3 × 1.3 mm, slice thickness = 7 mm (Figure 1). The approximately 10 second breath-hold CMR resulted in images with a temporal resolution of 22.5 ms. During AD measurement brachial blood pressure was measured using an oscillometric device (Schiller Magscreen, Schiller AG, Baar, Switzerland).

Left ventricular (LV) mass and function were measured using a true FISP sequence to acquire cine images in long axis planes (vertical long axis, horizontal long axis, LV outflow tract) followed by sequential short axis LV cine loops (8 mm slice thickness, 2 mm gap between slices) from the atrio-ventricular ring to the apex. LV mass index (LVMI) was calculated by normalising LV mass to the body surface area. Overall, scan time was approximately 30 minutes.

All CMR analyses were performed using conventional analysis software (Argus, Siemens, Erlangen, Germany). Cross sectional aortic contours were defined by manual planimetry throughout the cardiac cycle, with resulting aortic volume defined as the measured cross sectional area multiplied by the slice thickness. This calculation was performed automatically by the analysis program.

### Measurement of aortic distensibilty and volumetric arterial strain

Aortic distensibilty (AD) was calculated from change in aortic volume and simultaneous brachial blood pressure using the formula:

$$\text{Aortic distensibilty}=\frac{\left[{\left(\text{Aortic volume}\right)}_{\max}-{\left(\text{Aortic volume}\right)}_{\min}\right]}{\left[{\left(\text{Aortic volume}\right)}_{\min}*\text{pulse pressure}\right]}$$

where (Aortic volume)_max _and (Aortic volume)_min _are the maximal and minimal calculated aortic volumes during the cardiac cycle. Additionally, aortic volumetric arterial strain (VAS), which is non-pressure dependent, was calculated from the formula:

$$\text{VAS}=\frac{\left[{\left(\text{Aortic volume}\right)}_{\max}-{\left(\text{Aortic volume}\right)}_{\min}\right]}{\left[{\left(\text{Aortic volume}\right)}_{\min}\right]}$$

As a ratio of change in aortic volume, VAS does not have any units.

### Clinical data

All patients underwent conventional cardiovascular risk factor assessment including history, clinical examination, ECG as well as routine haematology, biochemical, and lipid profile. A history of ischaemic heart disease (IHD) was defined as previous clinically documented myocardial infarction/angina pectoris or previous coronary revascularisation procedure. Peripheral vascular disease was defined by clinically documented intermittent claudication with prior consultation by a vascular surgeon or the need for a previous peripheral vessel revascularisation procedure. Blood was drawn at the time of scanning for lipids and C – reactive protein, with other haematological and biochemical parameters (haemoglobin, dialysis adequacy, calcium-phosphate product) assessed from the mean of readings taken on the three consecutive months preceding the scan.

### Follow up

Follow up data was collected from date of the CMR scan using electronic patient records. Death and cardiovascular events (myocardial infarction, cerebrovascular event, coronary revascularisation and amputation for peripheral vascular disease) were collected as end points.

### Statistical methods

Correlations between cardiac dimensions, measures of arterial function and continuous clinical variables were assessed with Pearson and Spearman co-efficients. Differences between groups were tested by student's t-test and Mann-Whitney-U test. Measures of vascular function were compared between patients who died or had a vascular event during the follow up period with these measures divided into tertiles or quartiles and subjected to an unadjusted survival analysis by the Kaplan-Meier method. Statistical significance was determined by the log-rank test. Cox survival analysis was performed to assess the influence of multiple variables on outcome. Variables identified as possibly influential on outcome by univariate analysis were then entered into a forward stepwise regression model. As exponential values logarithmic transformation was used for AD and VAS values to simplify interpretation of values in the regression analyses. Analyses were performed using SPSS 13.0 software package (SPSS Inc., Chicago, IL., USA).

## Results

### Subjects

Patient demographics at the time of scan are shown in Table 1. 144 patients had aortic VAS measurement and 122 patients had measurement of AD available for analysis. 22 patients did not have blood pressure readings to calculate AD (either due to bilateral arteriovenous fistulae used as vascular access for dialysis making recording impossible, or due to blood pressure being recorded non-synchronously with the AD trace).

**Table 1:** Background demographics of patients studied

Number	144	
Age	51.5	(11.2)
Male (%)	90	(62.5)
Height (m)	169.5	(9.8)
Weight (kg)	75.9	(16.1)
On dialysis (%)	110	(76.4)
Haemodialysis	61	(42.4)
Peritoneal dialysis	49	(34.0)
RRT time (months)	6.0	(42.0)
Past history of IHD (%)	24	(16.7)
Diabetes (%)	46	(31.9)
Smoker (%)		
Never	83	(57.6)
Current	38	(26.4)
Ex	23	(16.0)
SBP (mmHg)	140.2	(24.2)
DBP (mmHg)	82.9	(12.8)
Cross sectional aortic volume (mL)	5.0	(2.0)
Aortic distensibilty (x 10^-3 ^mmHg^-1^)	2.4	(2.0)
Aortic volumetric arterial strain	0.13	(0.09)

Results are show as mean with standard deviation in parenthesis or number with percentage in parenthesis as appropriate, except for RRT time and measures of vascular function which are displayed as median and inter quartile range.

### Clinical correlates of aortic distensibilty and volumetric arterial strain

All scans were analysed by a single observer (P.B.M). No significant differences in patient demographics or vascular function were exhibited between patients with advanced renal failure not yet on dialysis therapy (34 patients; 24.6%) and those established on dialysis (110 patients) and therefore patients were combined as a single group. AD and VAS demonstrated a negative correlation with age (AD R = -0.44, p < 0.001, VAS R = -0.44, p < 0.001).

There were no significant correlations between haemoglobin, dialysis adequacy (urea reduction ratio in haemodialysis or creatinine clearance in peritoneal dialysis), time on dialysis, lipid parameters, C-reactive protein, calcium, phosphate or calcium phosphate product and AD or VAS. There were no significant differences in AD or VAS between genders. AD and VAS were reduced in patients with diabetes mellitus (diabetics – median AD 1.8 × 10^-3 ^vs. non-diabetics 2.8 × 10^-3 ^mmHg^-1^, p = 0.001; VAS 0.11 vs. 0.15, p = 0.001), patients with a history of ischaemic heart disease (median AD 1.8 × 10^-3 ^vs. 2.6 × 10^-3 ^mmHg^-1^, p = 0.028; VAS 0.11 vs. 0.15, p = 0.009) or peripheral vascular disease (median aortic distensibilty 1.2 × 10^-3 ^vs. 1.8 × 10^-3 ^mmHg^-1^, p = 0.005; VAS 0.14 vs. 0.07, p = 0.015). No significant differences in AD or VAS were demonstrated between patients treated with statins or requiring antihypertensive therapy. There were no significant differences between AD between dialysis modalities. 58 patients (40.3%) had a functioning arteriovenous fistula or graft at the time of scanning. There were no significant differences in AD or VAS between patients with an arteriovenous fistula or graft and those without.

Weak but statistically significant negative correlations were demonstrated between AD and LVMI (R = -0.21, p = 0.021) and end systolic volume (R = -0.18, p = 0.048) but no other LV dimension. Aortic VAS correlated with markers of cardiac function – left ventricular ejection fraction (R = 0.23, p = 0.006) and stroke volume (0.19, p = 0.024).

### Relationship between CMR measures of vascular function on survival and combined mortality and cardiovascular events

**Figure 2:**
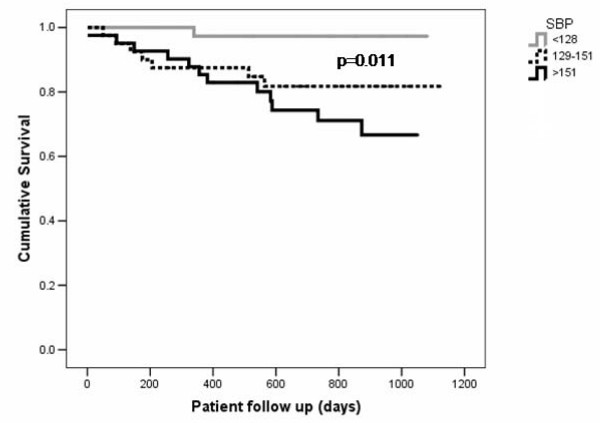
**Kaplan-Meier survival curves for all cause mortality with patients stratified by systolic blood pressure tertile**.

**Figure 3:**
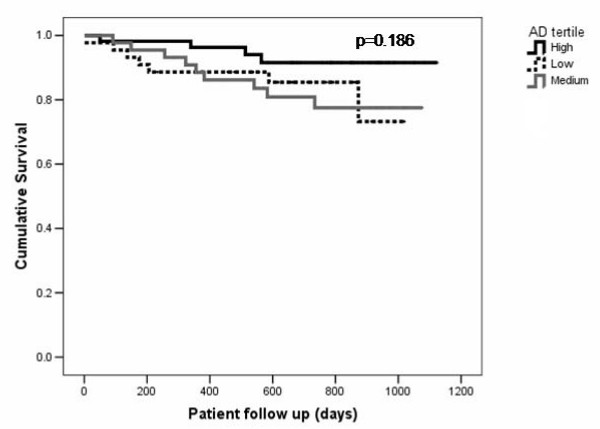
Kaplan-Meier survival curves for all cause mortality with patients stratified by aortic distensibilty tertile.

Follow up data were available for all 144 patients. Overall survival data was analysed for the whole cohort including analyses of the relationship between VAS and outcome. For analyses of the relationship between AD and outcome, only the 120 patients who only results of AD available were studied. The median follow up period was 719 days (interquartile range 375 days). There were 20 deaths, giving an overall mean death rate of 76.2 per 1000 patient years. Patients who died had significantly lower AD than those who where alive at the end of follow up. There were no significant differences in VAS between survivors and patients who died. Patients who died had significantly higher systolic and pulse pressure than survivors (Figures 2, 3), but there were no significant differences in diastolic blood pressure (Table 2).

**Table 2:** Demographic data for patients who were alive and dead at the end of the follow up period

	**Alive**		**Dead**		**p value**
Number	124	(86.1)	20	(13.9)	
Age	51.5	(11.3)	51.9	(11.1)	0.876
Male (%)	80	(64.5)	10	(50.0)	0.213
On dialysis (%)	92	(74.2)	18	(90.0)	0.122
RRT time (months)	6.0	(26.0)	36.0	(107.0)	0.023
Past history of IHD (%)	19	(15.3)	5	(25.0)	0.281
Diabetes (%)	34	(27.4)	12	(60.0)	0.004
Smoker (%)					
Never	73	(58.9)	10	(50.0)	
Current	30	(24.2)	8	(40.0)	0.304
Ex	21	(16.9)	2	(10.0)	
SBP (mmHg)	137.3	(23.6)	156.3	(21.4)	0.001
DBP (mmHg)	82.1	(12.9)	87.4	(11.7)	0.092
PP (mmHg)	55.20	(17.1)	69.0	(17.1)	0.001
Cross sectional aortic volume (mL)	5.0	(1.9)	5.1	(2.3)	0.634
Aortic distensibilty (x 10^-3 ^mmHg^-1^)	2.4	(2.0)	2.1	(2.1)	0.009
Aortic volumetric arterial strain	0.12	(0.09)	0.15	(0.10)	0.176

Results are show as mean with standard deviation in parenthesis or number with percentage in parenthesis as appropriate except for RRT time and measures of vascular function which are displayed as median and inter quartile range. Tests of significance are t-test and Chi-squared between groups, except for measures of vascular function where Mann-Whitney-U was used.

**Figure 4:**
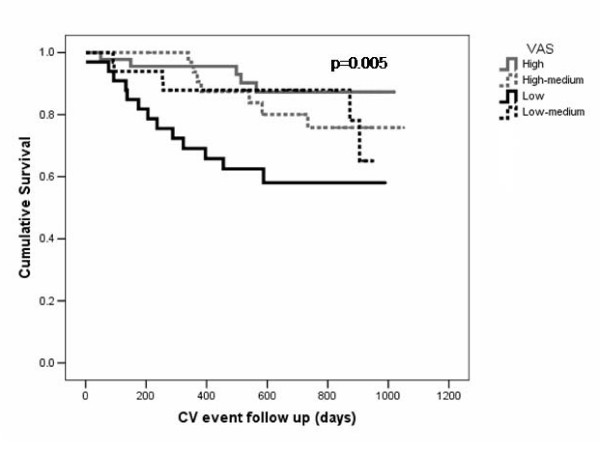
Kaplan-Meier survival curves for survival to either CV end point or death with patients stratified by aortic VAS quartile.

During the follow up period there were an additional 12 non-fatal CV events (five patients had a myocardial infarction; four patients underwent coronary revascularisation, two patients undergoing amputation for peripheral vascular disease and one patient had a cerebrovascular event). These patients had significantly lower AD and VAS than those who remained event free (median AD in patients with CV events 1.5 × 10^-3 ^vs. 2.8 × 10^-3 ^mmHg^-1^, p = 0.006; VAS 0.12 vs. 0.14, p = 0.012; Figure 4). There were no significant difference in blood pressure between patients who remained event free and patients who had a non fatal CV event.

Combining death with non fatal CV events as a combined CV end point, patients who reached this combined end point had significantly lower AD (median AD 1.7 × 10^-3 ^vs. 2.7 × 10^-3 ^mmHg^-1^, p < 0.001), VAS (0.12 vs. 0.14, p = 0.006) and higher systolic blood pressure (mean 152.9 vs. 136.5 mmHg, p = 0.001) and pulse pressure (mean 67.0 vs. 54.5 mmHg, p = 0.001) than those who did not. There were no significant differences in diastolic blood pressure (mean 85.9 vs. 82.0 mmHg, p = 0.154) between those who reached the combined end point and those who did not.

In a Cox forward stepwise regression model assessing patient survival; diabetes, systolic blood pressure and AD were significant independent predictors of patient survival (Table 3). In a similar analysis assessing the combined CV end point of death or a vascular event; diabetes, AD and aortic VAS were significant predictors of events (Table 4). Due to their close interdependence, blood pressure variables and AD were entered individually into the each model to determine their influence. Similarly either AD or VAS was entered individually to the model but not together.

**Table 3:** Patient survival

	**Univariate analysis**			**Multivariate analysis**		
**Variable**	**HR**	**(95.0% CI)**	**p**	**HR**	**(95.0% CI)**	**p**
Diabetes	3.031	(1.036, 8.866)	0.043	4.214	(1.631, 10.886)	0.003
SBP (mmHg)	1.015	(0.992, 1.038)	0.197	1.022	(1.000, 1.044)	0.049
Log aortic distensibilty	0.072	(0.008, 0.694)	0.023	0.135	(0.019, 0.948)	0.044
Log aortic VAS	0.107	(0.007, 1.732)	0.116			
Duration RRT (days)	1.000	(1.000, 1.000)	0.421			
Age (years)	1.015	(0.969, 1.063)	0.526			
Gender (ref male)	0.833	(0.312, 2.226)	0.716			
Haemoglobin (g/dL)	0.910	(0.670, 1.236)	0.547			
Albumin (g/L)	0.942	(0.839, 1.058)	0.311			

Cox survival analysis for patient survival. Because of their close correlation SBP aortic distensibilty and aortic VAS were entered separately into the model. Results for other variables are shown with systolic blood pressure in the model

**Table 4:** Combined CV event and death survival

	**Univariate analysis**			**Multivariate analysis**		
**Variable**	**HR**	**(95.0% CI)**	**p**	**HR**	**(95.0% CI)**	**p**
Diabetes	2.989	(1.276 7.004)	0.012	3.575	(1.669 7.655)	0.001
Log aortic distensibilty	0.052	(0.009 0.319)	0.001	0.066	(0.013 0.347)	0.001
Log aortic VAS	0.021	(0.002 0.206)	0.001	0.026	(0.004 0.175)	<0.001
Duration RRT (days)	1.000	(1.000 1.000)	0.560			
SBP (mmHg)	1.014	(0.995 1.032)	0.142			
Age (years)	1.023	(0.984 1.062)	0.248			
Gender (ref male)	0.827	(0.368 1.860)	0.646			
Haemoglobin (g/dL)	0.958	(0.748 1.225)	0.731			
Albumin (g/L)	0.984	(0.896 1.080)	0.728			

Cox survival analysis for patient survival to either death or cardiovascular event. Because of their close correlation SBP aortic distensibilty and aortic VAS were entered separately into the model. Results for other variables are shown with systolic blood pressure in the model.

## Discussion

This is the first study to examine the relationship between AD assessed with CMR and outcome in ESRD. Patients with ESRD have previously been demonstrated to have increased aortic stiffness compared to controls, represented by reduced AD and VAS with associated vessel dilatation[6]. In keeping with the relationship between arteriosclerosis and advancing age, there was a significant relationship between reduced AD and VAS and age. AD and VAS were both lower in patients at highest CV risk, i.e. those with diabetes, ischaemic heart disease and/or peripheral vascular disease. No other factors such as drug therapy, adequacy of dialysis, duration of renal replacement therapy, dialysis modality or any laboratory parameter appeared to be significantly associated with AD or VAS.

One major potential limitation of our study is that calculation of AD is based on cross-sectional volume of the vessel wall and pulse pressure. For practical reasons, direct aortic blood pressure measurement has been substituted by non-invasive indirect brachial blood pressure. Brachial blood pressure is not entirely representative of central haemodynamics and our methods do not permit assessment of the phenomenon of central to peripheral systolic and pulse pressure amplification, as can be measured with tonometry. It is difficult to dissociate any clinical effect associated with changes in AD from that due directly to pulse pressure, which is a well described predictor of outcome in ESRD. Nonetheless, as it is otherwise impossible to assess aortic stiffness without documentation of pressure within the vessel lumen, the approach used in this study has been used widely[7,9-11]. For this reason, aortic VAS, the fractional increase in aortic volume during the cardiac cycle was also used. This does not depend on blood pressure variables and in this study displayed similar relationships with clinical variables and outcome. However, there is limited literature to support its use. Other studies have demonstrated that PWV can be measured with CMR. There are advantages to using measuring PWV compared to AD as it is load-independent, negating the necessity for blood pressure measurements. However, at the time of initiation of this study, there were few studies in any patient group other than healthy volunteers[10,12], and unfortunately CMR derived PWV was not measured in this study cohort.

The relationship between these markers of aortic function and LV dimensions show a weak but significant relationship between increased aortic stiffness and increasing LVMI and end systolic volume, suggesting that reduced AD may increase LV wall tension and hence cardiac hypertrophy. Aortic VAS, which is independent of blood pressure, correlated with markers of systolic function, namely ejection function and stroke volume, suggesting that in the failing heart where systolic blood pressure is lower, increased arterial stiffness remains deleterious to ventricular performance.

A variety of studies have addressed the role of AD as a marker of either CV risk or relating AD to cardiac performance in other groups at high risk of CV disease. Data relating CMR measures of arterial function to long term outcome are scarce. In otherwise healthy individuals, obese subjects have been shown by CMR to have increased aortic cross sectional area and decreased aortic elasticity[13,14]. AD is reduced in patients with heart failure and correlates with exercise capacity[9]. One study in hypertensive patients has demonstrated that AD increases following treatment with nicardipine or alacepril but not trichlormethiazide, independent of changes in pulse pressure[15]. More recently, in patients with non diabetic CKD, AD has been shown to correlate with GFR demonstrating that CMR can quantify the impact of reduced kidney function on vascular function in early CKD[7]. Additionally, endothelial function can be studied using CMR to by assessing cross sectional flow mediated dilation of the brachial artery[16].

Independent predictors of mortality during the follow up period were diabetes, AD and systolic blood pressure. Due to their close interdependence, systolic blood pressure and aortic distensibilty could not be independently assessed. When a combined end point of death, non-fatal myocardial infarction, cardiac revascularisation, amputation for peripheral vascular disease and cerebrovascular event was used, only diabetes and AD or VAS were independent predictors of events. Surprisingly, age was not a predictor of outcome, suggesting that since age and AD were closely correlated, vascular, rather than temporal, aging is a more important determinant of survival. In keeping with other studies, time on renal replacement therapy was significantly longer in those patients who died[4], but this was not independently associated with outcome. Therefore in this patient group, AD was a predictor of mortality and/or vascular events independent of age and dialysis vintage.

Haemodynamic factors which promote arterial remodelling present in ESRD, although not specific to kidney disease, include age and blood pressure. Factors specific to ESRD include arteriovenous shunts and chronic volume overload[17]. Increased blood flow through a shunt promotes arterial remodelling although may not lead to detectible changes in measures of vascular function[18]. We could find no difference in AD or VAS in patients with an arteriovenous shunt and those without. As novel marker of increased CV risk, no data exist on the natural history of progression of AD, or whether it can be improved by therapeutic intervention. Our results suggest that as there was no significant difference between those CKD 5 patients on dialysis compared to pre-dialysis patients, the major derangement in vascular function occurs earlier during the progression of CKD. One assumes that reduced AD is a consequence of arterial calcification[19], although we cannot confirm this as CMR is unable to display calcific lesions due to their absence of water content.

A large number of studies have been performed to assess the determinants of arterial stiffness in ESRD, and its relation to long term survival, using either incremental elastic modulus, PWV or augmentation index as markers of arterial stiffness. In keeping with our results, factors associated with increased arterial stiffness include age, diabetes, and systolic blood pressure (or pulse pressure) [20-22]. Other factors associated with arterial stiffness include serum calcium or the presence of inflammation[22]. Surprisingly, these factors were not predictors of vascular function in our study. Arterial stiffness has been repeatedly demonstrated to be an independent predictor of all cause and cardiovascular mortality in haemodialysis patients[3,4,23].

Aside from the issue of use of brachial blood pressure to calculate central haemodynamics, our study has some other limitations. Although direct comparison between CMR and calcification is not possible, CMR has emerged as a technique for imaging atheroma. A direct comparison between vascular function and dark blood imaging looking at aortic wall thickness and atheroma would be of interest but unfortunately was not performed. Measurement of AD is still a relatively novel technique and its widespread reproducibility is yet to be established. In house blinded analysis has demonstrated an inter-observer variability of 4.6%, although this needs to be reproduced in other centres, particularly as there are potential sources of error in both data acquisition and analysis as well as in blood pressure measurements. As we have previously used CMR with late gadolinium enhancement (LGE) to analyse myocardial tissue composition in ESRD[5], it would have been of interest to compare myocardial tissue abnormalities with vascular function. However, as a link between gadolinium and nephrogenic systemic fibrosis has emerged, this comparison is impossible[24]. It is unfortunate that it was not possible to acquire brachial blood pressure on every patient due to some patients having multiple vascular procedures on both upper limbs. Finally, CMR technique is relatively expensive and time consuming to analyse. With future development of automated analysis software, faster analysis of AD will be possible.

## Conclusion

This study has demonstrated that vascular function can be measured using CMR in ESRD. Increased aortic stiffness, indicated by reduced AD and reduced VAS are associated with risk factors for CV disease. Both AD and VAS were independent predictors of combined vascular events and mortality. AD predicted all cause mortality. Therefore, CMR offers a novel non-invasive tool to assess vascular function in patients ESRD. AD and VAS are potential targets for therapeutic intervention to reduce cardiovascular risk in ESRD.

## Competing interests

The authors declare that they have no competing interests.

## Authors' contributions

Guarantor of integrity of entire study PBM, study concepts/study design PBM, AD, KGB, TS, JEF, HJD, AGJ data collection and analysis PBM, AD, KGB, RKP, RAPW, TS, JE, AGJ, statistical analysis PBM, RKP, AGJ, manuscript drafting or manuscript revision for important intellectual content, all authors; manuscript final version approval, all authors.
